# Role of protein kinase C and NF-*κ*B in proteolysis-inducing factor-induced proteasome expression in C_2_C_12_ myotubes

**DOI:** 10.1038/sj.bjc.6601767

**Published:** 2004-04-06

**Authors:** H J Smith, S M Wyke, M J Tisdale

**Affiliations:** 1 Pharmaceutical Sciences Research Institute, Aston University, Birmingham, B4 7ET, UK

**Keywords:** Proteolysis-inducing factor, protein kinase C, nuclear factor-*κ*B, proteasome expression

## Abstract

Proteolysis-inducing factor (PIF) is a sulphated glycoprotein produced by cachexia-inducing tumours, which initiates muscle protein degradation through an increased expression of the ubiquitin–proteasome proteolytic pathway. The role of kinase C (PKC) in PIF-induced proteasome expression has been studied in murine myotubes as a surrogate model of skeletal muscle. Proteasome expression induced by PIF was attenuated by 4*α*-phorbol 12-myristate 13-acetate (100 nM) and by the PKC inhibitors Ro31-8220 (10 *μ*M), staurosporine (300 nM), calphostin C (300 nM) and Gö 6976 (200 *μ*M). Proteolysis-inducing factor-induced activation of PKC_*α*_, with translocation from the cytosol to the membrane at the same concentration as that inducing proteasome expression, and this effect was attenuated by calphostin C. Myotubes transfected with a constitutively active PKC_*α*_ (*p*CO_2_) showed increased expression of proteasome activity, and a longer time course, compared with their wild-type counterparts. In contrast, myotubes transfected with a dominant-negative PKC_*α*_ (pKS1), which showed no activation of PKC_*α*_ in response to PIF, exhibited no increase in proteasome activity at any time point. Proteolysis-inducing factor-induced proteasome expression has been suggested to involve the transcription factor nuclear factor-*κ*B (NF-*κ*B), which may be activated through PKC. Proteolysis-inducing factor induced a decrease in cytosolic I-*κ*B*α* and an increase in nuclear binding of NF-*κ*B in *p*CO_2_, but not in pKS1, and the effect in wild-type cells was attenuated by calphostin C, confirming that it was mediated through PKC. This suggests that PKC may be involved in the phosphorylation and degradation of I-*κ*B*α*, induced by PIF, necessary for the release of NF-*κ*B from its inactive cytosolic complex.

Loss of skeletal muscle in cancer cachexia results in asthenia, immobility and eventually death through impairment of respiratory function. Nutritional supplementation alone is unable to reverse this wasting process (Evans *et al*, 1985), suggesting that the balance between protein synthesis and degradation is impaired, especially since visceral protein reserves are preserved and may even increase (Fearon, 1992). Thus, while protein synthesis in skeletal muscle of cachectic patients is impaired (Lundholm *et al*, 1976), there is also an increase in protein degradation (Lundholm *et al*, 1982). An increased activity of the ubiquitin–proteasome proteolytic pathway is considered to be the major factor for this increased protein degradation (Williams *et al*, 1999).

Tumour production of a sulphated glycoprotein called proteolysis-inducing factor (PIF) may be responsible for the progressive loss of skeletal muscle in cancer cachexia (Todorov *et al*, 1996). Proteolysis-inducing factor is only produced by cachexia-inducing tumours, and when purified and administered to mice it induces a specific loss of skeletal muscle, while visceral protein is maintained or even increased, as in cancer cachexia (Lorite *et al*, 1998). Using a surrogate model of skeletal muscle, PIF was shown to inhibit protein synthesis and increase protein degradation (Smith *et al*, 1999). The increased protein degradation was shown to arise from an increased expression of the ubiquitin–proteasome proteolytic pathway (Lorite *et al*, 2001).

Protein degradation induced by PIF was accompanied by an increased release of arachidonic acid from membrane phospholipids and its subsequent metabolism to 15-hydroxyeicosatetraenoic acid (15-HETE), which was shown to be related to protein catabolism. Both arachidonic acid and lipoxygenase metabolites have been shown to activate protein kinase C (PKC) (Fan *et al*, 1990), which may play a role in PIF-induced proteasome expression. Phosphorylation of proteasome subunits may be important in the regulation of proteasome activity, since some proteasome subunits have potential tyrosine (Tanaka *et al*, 1990) and serine/threonine (Heinemeyer *et al*, 1994) phosphorylation sites and dephosphorylation by acid phosphatase have been shown to lower proteasome activity significantly (Mason *et al*, 1996). At least one subunit (S4) has several potential PKC phosphorylation sites (Dubiel *et al*, 1992). Alternatively, PKC may play a role in activation of the transcription factor nuclear factor-*κ*B (NF-*κ*B), which has been shown to be involved in the production of interleukin-8 (IL-8), IL-6, C-reactive protein and ICAM-1 in liver cells (Watchorn *et al*, 2001), and may also be involved in PIF-induced proteasome expression (Whitehouse and Tisdale, 2003). Protein kinase C has been suggested as being an upstream activator of the I-*κ*B kinase complex (IKK) (Lallena *et al*, 1999; Trushin *et al*, 1999; Vertegaal *et al*, 2000) leading to I-*κ*B*α* phosphorylation and ubiquitination and the subsequent processing of the 26S proteasome followed by the translocation of NF-*κ*B into the nucleus.

The present study investigates the role of PKC in PIF-induced proteasome expression and its relationship to activation of NF-*κ*B in C_2_C_12_ murine myotubes.

## MATERIALS AND METHODS

### Materials

Foetal calf serum (FCS), horse serum (HS) and Dulbecco's modified Eagle's medium (DMEM) were purchased from Invitrogen (Paisley, Scotland). Mouse monoclonal antibodies to proteasome 20S *α*-subunits were from Affiniti Research Products (Exeter, UK). Rabbit polyclonal antisera to ubiquitin conjugating enzyme (E2_14k_) were a gift from Dr Simon Wing, McGill University (Montreal, Canada). Rabbit polyclonal antisera to murine I-*κ*B*α*, *β*-tubulin and PKC_*α*_ were from Calbiochem (Herts, UK) as were PMA, Ro31-8220, calphostin C, staurosporine and Gö 6976. Rabbit polyclonal antisera to mouse actin were from Sigma-Aldridge (Dorset, UK). Peroxidase-conjugated goat antirabbit and rabbit antimouse secondary antibodies were from Dako Ltd (Cambridge, UK). Hybond™ nitrocellulose membranes and enhanced chemiluminescence (ECL) were from Amersham Life Science Products (Bucks, UK). Electrophoretic-mobility shift (EMSA) gel shift assay kits were from Panomics (California, USA). *Escherichia coli* DH5*α* cells were from Invitrogen (Paisley, Scotland). Constitutively active and mutant plasmids of PKC_*α*_ were a gift from Prof. Peter Parker (Cancer Research, UK). The insert A25E PKC_*α*_ is constitutively active due to a deletion of amino acids 22–28 in the N-terminal region and is expressed via the *p*CO_2_ vector (Pears *et al*, 1990). Protein kinase C *α* (T/A)_3_ is a dominant-negative mutant expressed in pKS1 (Bornancin and Parker, 1996). Plasmid DNA was purified using the WIZARD® PureFection purification system (Promega, Southampton, UK) according to the manufacturer's protocol. Primers for PCR analysis were from MWG Biotech (Ebersberg, Germany). GeneJuice™ for transfection studies was purchased from Calbiochem (Herts, UK).

### Transformation of bacteria

*E. coli* DH5_*α*_ were transformed with both constitutively active and mutant PKC_*α*_ using heat shock, and selected with ampicillin (100 *μ*g ml^−1^). Positive clones were identified using primers with homology to bovine PKC (forward 5′-CAC CTG TGA TAT GAA CGT GC-3′ reverse 5′-GAA GTT GAA GTC CGT GAG C-3′). The product was about 600 bp as determined on a 2% agarose gel. Plasmid DNA was extracted from positive colonies grown overnight in an LB medium containing ampicillin (100 *μ*g ml^−1^).

### Purification of PIF

PIF was purified from solid MAC16 tumours excised from mice with a weight loss between 20 and 25% as previously described (Todorov *et al*, 1996; Whitehouse and Tisdale, 2003). Tumours were homogenised in 10 mM Tris-HCl, pH 8.0, containing 0.5 mM phenylmethylsulphonyl fluoride, 0.5 mM EGTA and 1 mM dithiothreitol at a concentration of 5 ml g^−1^ tumour. The supernatant obtained after addition of ammonium sulphate (40% w v^−1^) was subjected to affinity chromatography using anti-PIF monoclonal antibody coupled to a solid matrix. The immunogenic fractions were concentrated and used for further studies.

### Myogenic cell culture and transfection

The C_2_C_12_ myoblast cell line was grown in DMEM supplemented with 10% FCS plus 1% penicillin and streptomycin under an atmosphere of 10% CO_2_ in air. Transfection was carried out on cells at 50% confluency using GeneJuice™ transfection reagent, according to the manufacturer's protocol and selected by resistance to ampicillin (5 g l^−1^). Transfected myoblasts were stimulated to differentiate by replacing the growth medium with DMEM supplemented with 2% HS, when the cells reached confluence. Differentiation was allowed to continue for 5–9 days until myotubes were clearly visible, and used for the experiments described in results.

### Measurement of proteasome ‘chymotrypsin-like activity

‘Chymotrypsin-like’ enzyme activity was determined fluorimetrically by the method of Orino *et al* (1991) as previously described (Lorite *et al*, 2001). Myotubes were washed with ice-cold phosphate-buffered saline (PBS) and sonicated in 20 mM Tris-HCl, pH 7.5, 2 mM ATP, 5 mM MgCl_2_ and 1 mM dithiothreitol at 4°C. The supernatant formed by centrifugation at 18 000 **g** for 10 min was used to measure the ‘chymotrypsin-like’ enzyme activity by the release of aminomethyl coumarin (AMC) from the fluorogenic peptide succinyl-LLVY-AMC (0.1 mM). Activity was measured in the presence and absence of the specific proteasome inhibitor lactacystin (10 *μ*M). Only lactacystin-suppressible activity was considered to be proteasome specific.

### Western blot analysis

Cytoplasmic proteins, obtained from the above assay, were also used for Western blotting, while the pellet was dissolved in sonicating buffer containing 0.1% Nonidet P40 and used as a source of cell membranes. Both extracts were loaded at 2–5 *μ*g protein and resolved on 10% sodium dodecylsulphate: polyacrylamide gels and transferred to Hybond™ nitrocellulose membrane. Membranes were blocked with 5% Marvel in PBS. The primary antibodies for PKC_*α*_, E2_14k_ and *β*-tubulin were used at a dilution of 1 : 100, while antibodies for I-*κ*B*α* were at 1 : 1000 and 20S proteasome *α*-subunits at 1 : 1500. The secondary antibodies were used at a dilution of 1 : 2000. Incubation was carried out for 2 h at room temperature, and development was by ECL.

### Electrophoresis mobility shift assay

DNA-binding proteins were extracted from myotubes by the method of Andrews and Faller (1991), which utilises hypotonic lysis followed by high salt extraction of nuclei. The EMSA-binding assay was carried out using a Panomics EMSA ‘gel shift’ kit according to the manufacturer's instructions.

### Statistical analysis

Differences as means between groups was determined by one-way ANOVA followed by Tukey–Kramer multiple comparison test.

## RESULTS

**Figure 1:**
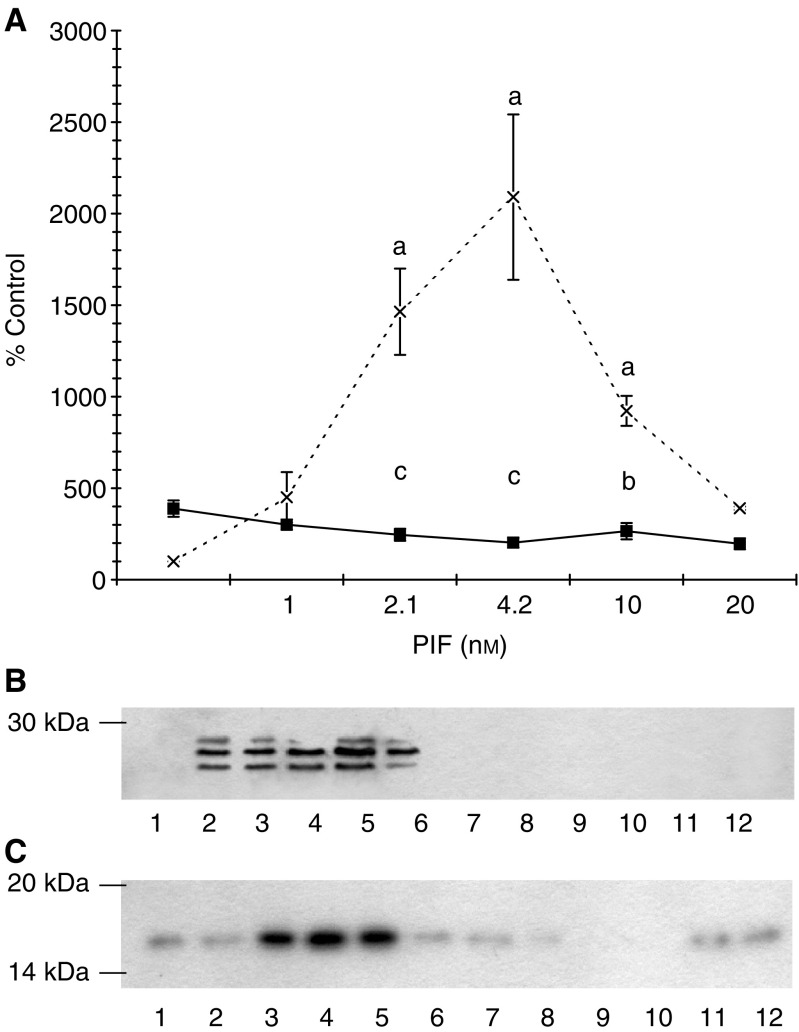
(**A**) Effect of PMA on PIF-induced chymotrypsin-like enzyme activity in murine myotubes. Cells were incubated either with PIF alone (×) or in the presence of PMA (100 nM), added 2 h prior to PIF (▪), and the chymotrypsin-like enzyme activity was determined after 24 h, as described in Materials and methods. The experiment was repeated three times (*n*=9). Differences from control are indicated as ^a^*P*<0.001, while differences from cells incubated in the presence of PIF alone are shown as ^b^*P*<0.05 and ^c^*P*<0.001. (**B**) Western blot of the effect of PMA on proteasome 20S *α*-subunits and (**C**) E2_14k_ 24 h after addition of PIF. Cells were incubated with 0 (lanes 1 and 7), 1.0 (lanes 2 and 8), 2.1 (lanes 3 and 9), 4.2 (lanes 4 and 10), 10 (lanes 5 and 11) or 20 nM PIF (lanes 6 and 12) in the absence (lanes 1–6) or presence (lanes 7–12) of PMA (100 nM). A representative blot is shown and the experiment was repeated three times.

To evaluate the role of PKC in PIF-induced proteasome expression, the effect of excess 4*α*-phorbol 12-myristate 13-acetate (PMA) on ‘chymotrypsin-like’ enzyme activity, the predominant proteolytic activity of the proteasome (Figure 1A

), and on expression of proteasome 20S_*α*_ subunits (Figure 1B) and the ubiquitin-conjugating enzyme (E2_14k_) (Figure 1C) was determined in C_2_C_12_ myotubes 24 h after PIF addition. Proteolysis-inducing factor produced an increase in ‘chymotrypsin-like’ enzyme activity, proteasome 20S_*α*_ subunits and E2_14k_ with a maximal effect between 2.1 and 10 nM, and this effect was completely attenuated in myotubes pretreated with PMA. These results suggest that PKC may be important in PIF-induced proteasome expression.

**Figure 2:**
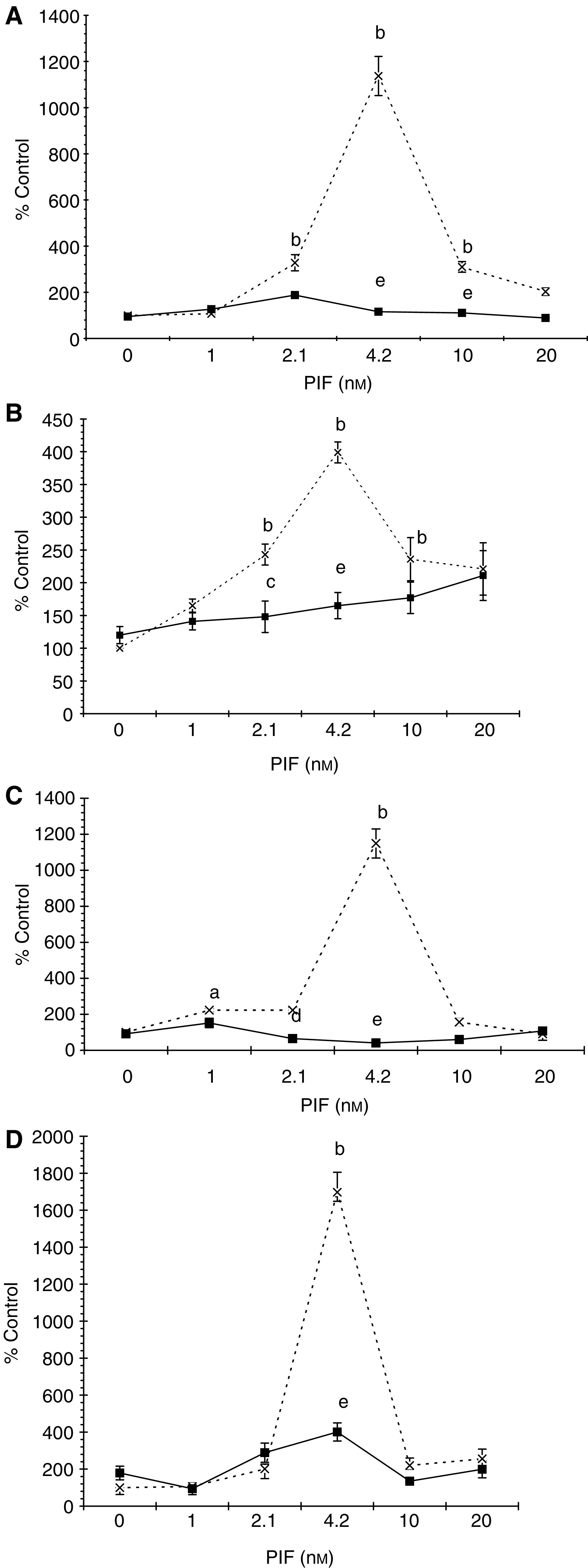
Effect of inhibitors of PKC on PIF-induced chymotrypsin-like enzyme activity. Myotubes were incubated with PIF alone (×) or with Ro 31-8220 (1 *μ*M) (**A**); staurosporine (300 nM) (**B**); calphostin C (300 nM) (**C**); or with Go 6976 (200 *μ*M) (**D**) added 2 h prior to PIF, and chymotrypsin-like enzyme activity was determined 24 h after addition of PIF. The experiment was repeated three times (*n*=9). Differences from control are indicated as ^a^*P*<0.05 and ^b^*P*<0.001, while differences from cells incubated in the presence of PIF alone are shown as ^c^*P*<0.05, ^d^*P*<0.01 and ^e^*P*<0.001.

**Figure 3:**
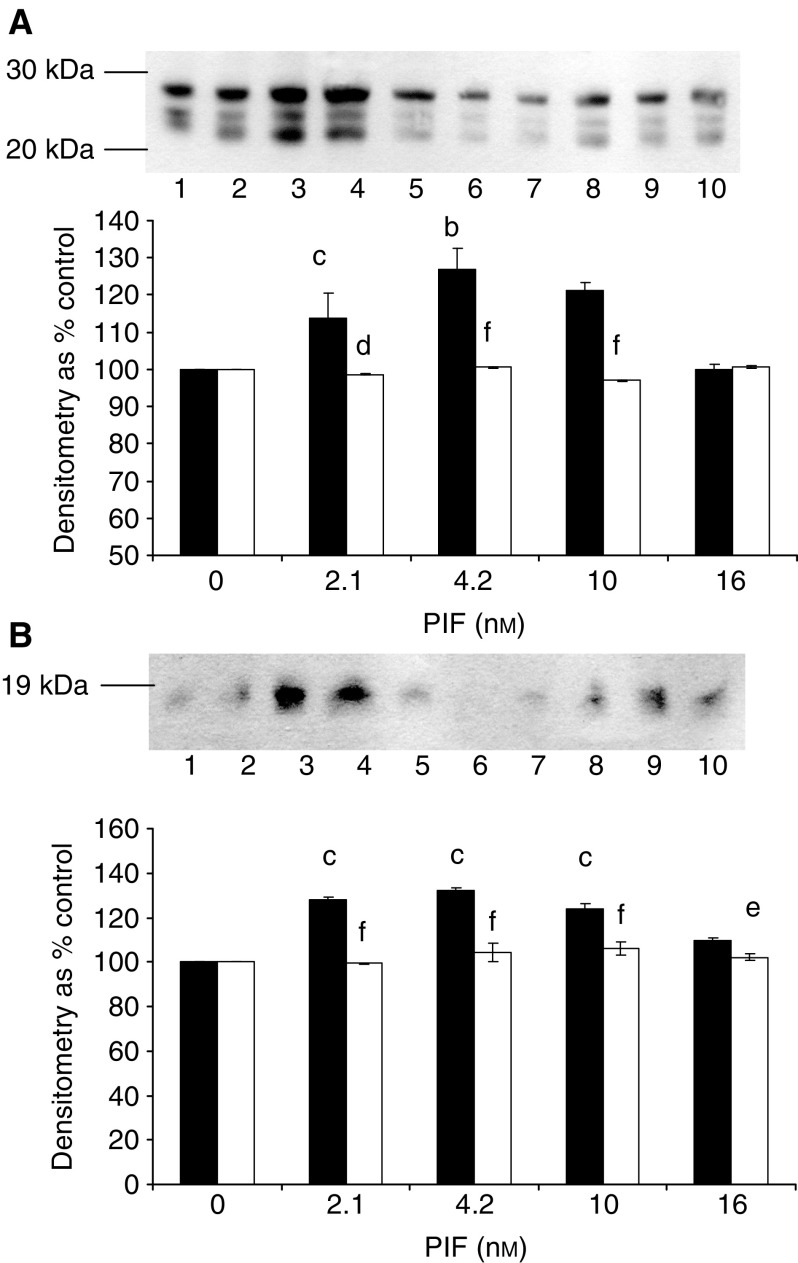
Western blot of the effect of calphostin C on 20S proteasome *α*-subunit expression (**A**) and E2_14k_ (**B**) in the presence of PIF. Cells were incubated with 0 (lanes 1 and 6), 2.1 (lanes 2 and 7), 4.2 (lanes 3 and 8), 10 (lanes 4 and 9) or 16.8 nM PIF (lanes 5 and 10) either alone (lanes 1–5) or in the presence of calphostin C (300 nM) and expression was determined after 24 h. A representative blot is shown and the densitometric analysis is based on three replicate blots. Values in the presence of PIF are indicated as ▪ and in the presence of PIF and calphostin C as □. The densitometric analysis of the 20S subunits represents the average of the two major bands. Differences from control are indicated as ^a^*P*<0.05, ^b^*P*<0.01 and ^c^*P*<0.001, while differences from the presence of PIF alone are shown as ^d^*P*<0.05, ^e^*P*<0.01 and ^f^*P*<0.001.

**Figure 4:**
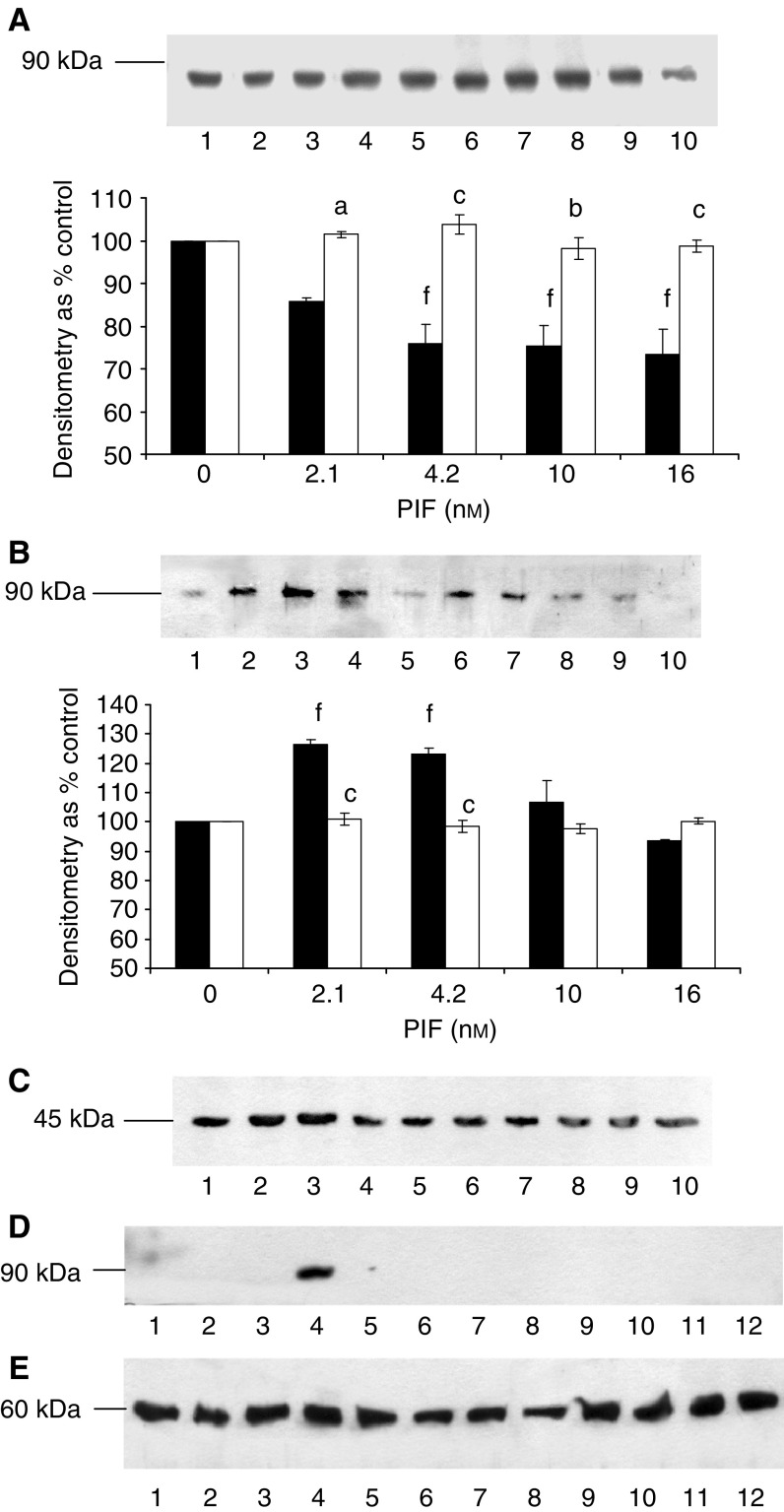
Effect of PIF on activation of PKC_*α*_ in murine myotubes in the absence or presence of calphostin C (**A**, **B**) or EPA (**C**). (**A**) Cytoplasmic and (**B**) Membrane-bound PKC_*α*_ after incubation with 0 (lanes 1 and 6), 2.1 (lanes 2 and 7), 4.2 (lanes 3 and 8), 10 (lanes 4 and 9) or 16.8 nM PIF (lanes 5 and 10) for 24 h in the absence (lanes 1–5) or presence (lanes 6–10) of calphostin C (300 nM). The densitometric analysis was based on three replicate blots, and values in the presence of PIF are shown as ▪ and in the presence of PIF and calphostin C as □. Differences from control are shown as ^c^*P*<0.001, while differences from PIF alone are shown as ^d^*P*<0.05, ^e^*P*<0.01 and ^f^*P*<0.001. (**C**) Actin loading control for the blots shown in (**A**, **B**). (**C**) Actin loading control for the blots shown in (**A**, **B**). (**D**) Effect of EPA (50 *μ*M) on membrane-bound PKC_*α*_ in the presence of PIF. Cells were loaded with 0 (lanes 1 and 7), 1.0 (lanes 2 and 8), 2.1 (lanes 3 and 9), 4.2 (lanes 4 and 10), 10 (lanes 5 and 11) or 20 nM PIF (lanes 6 and 12) either in the absence (lanes 1–6) or after 2 h pretreatment with EPA (50 *μ*M), and membrane-bound PKC_*α*_ was determined after 24 h. (**E**) *β*-tubulin loading control for the blot shown in (**D**).

To confirm a role for PKC in this process, the effect of Ro31-8220, a competitive and selective PKC inhibitor (Beltman *et al*, 1996), staurosporine, a broad-spectrum inhibitor of protein kinases (Couldwell *et al*, 1994), calphostin C, a highly specific inhibitor of PKC (Jarvis *et al*, 1994), and Gö 6976, which selectively inhibits PKC_*α*_ and *β*, isoenzymes (Wang *et al*, 1998), on the PIF-induced increase in ‘chymotrypsin-like’ enzyme activity was determined (Figure 2

). The PIF-induced enzyme activity was completely attenuated by 10 *μ*M Ro31-8220 (Figure 2A), 300 nM staurosporine (Figure 2B), 300 nM calphostin C (Figure 2C) and 200 *μ*M Gö 6976 (Figure 2D). In addition, calphostin C completely attenuated the PIF-induced increase in proteasome 20S *α*-subunit expression (Figure 3A

) and E2_14k_ (Figure 3B). Proteolysis-inducing factor induced a decrease in cytosolic PKC (Figure 4A

) and an increase in membrane-bound PKC_*α*_ (Figure 4B) at the same concentrations as those inducing proteasome expression (Figure 1) and this effect was attenuated by both calphostin C (Figure 4A and B) and eicosapentaenoic acid (EPA) (Figure 4D). These results confirm a role for PKC in PIF-induced proteasome expression, and suggest another mechanism by which EPA may attenuate PIF-induced protein degradation through inhibition of PKC.

**Figure 5:**
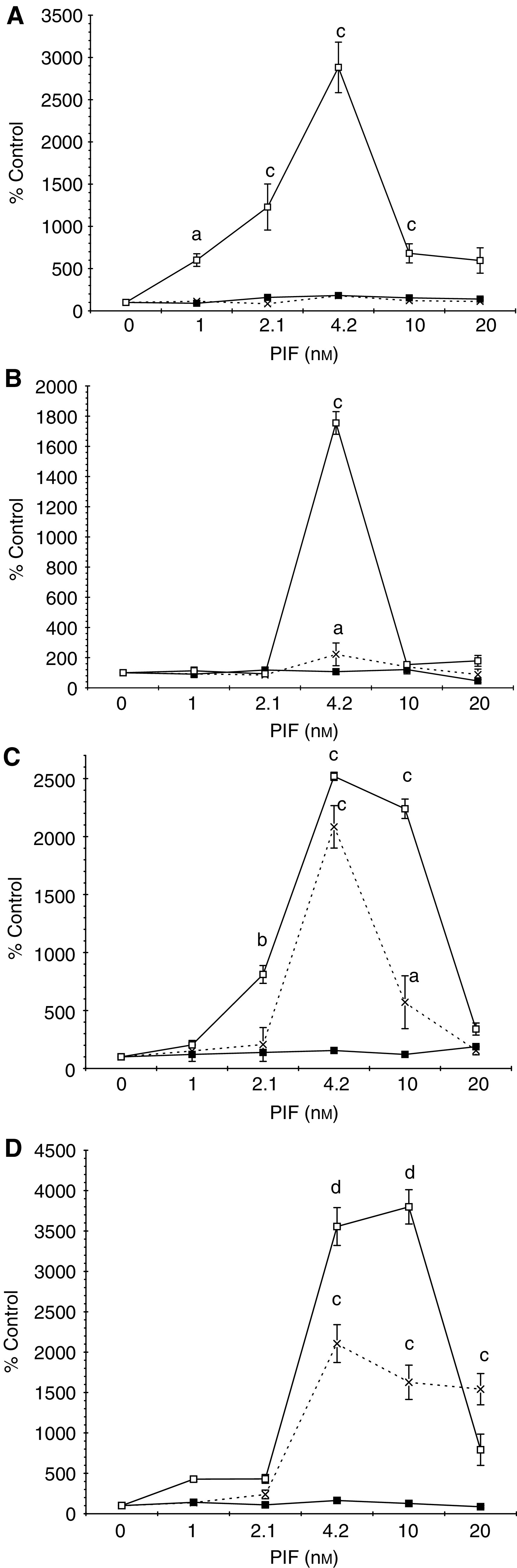
Time course for induction of chymotrypsin-like enzyme activity in wild-type myotubes (×) and in those transfected with constitutively active (*p*CO_2_ □) and mutant (pKS1 ▪) PKC_*α*_. Myotubes were treated with the indicated concentrations of PIF, and enzyme activity was determined at 3 h (**A**), 6 h (**B**), 24 h (**C**) and 48 h (**D**). The experiment was repeated three times (*n*=9). Differences from control or pKS1 are indicated as ^a^*P*<0.05, ^b^*P*<0.01 and ^c^*P*<0.005, while differences from wild-type myotubes are indicated as ^d^*P*<0.005.

**Figure 6:**
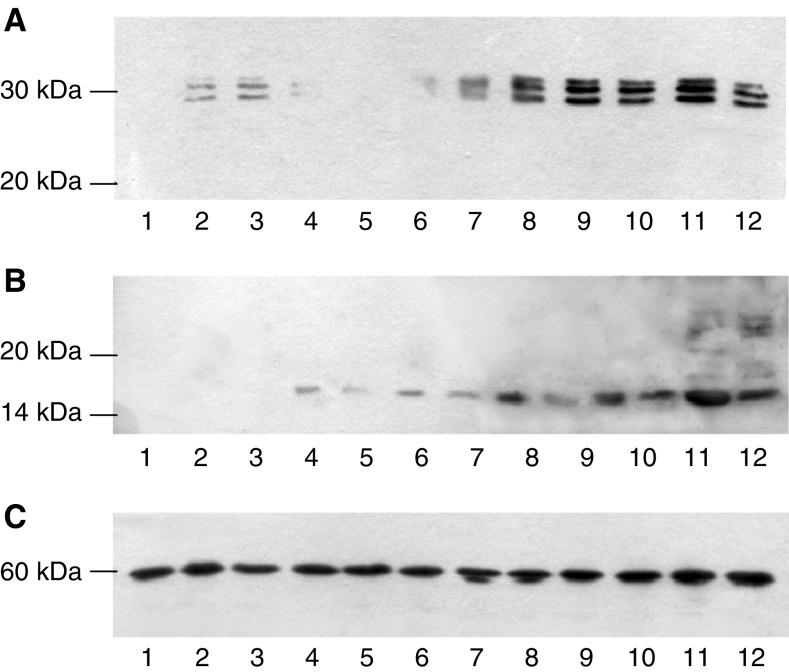
Western blot of the effect of PIF on 20S proteasome *α*-subunit expression (**A**) and E2_14k_ (**B**) in pKS1 (lanes 1–6) and *p*CO_2_ (lanes 7–12) after treatment with 0 (lanes 1 and 7), 1.0 (lanes 2 and 8), 2.1 (lanes 3 and 9), 4.2 (lanes 4 and 10), 10 (lanes 5 and 11) and 20 nM PIF (lanes 6 and 12) determined after 24 h. (**C**) *β*-tubulin loading control.

**Figure 7:**
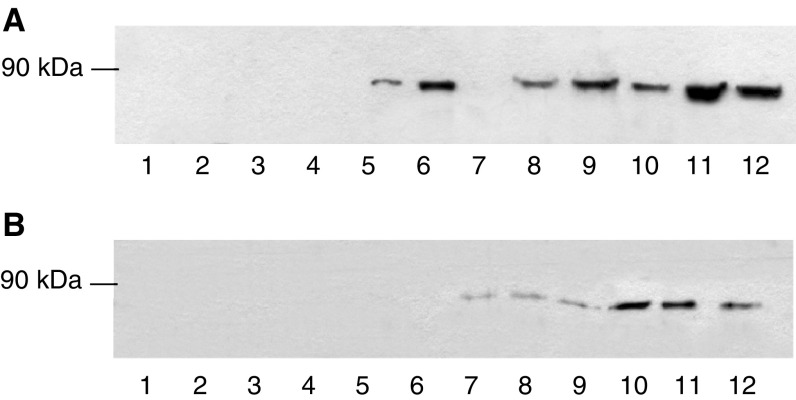
Western blot of the effect of PIF on cytoplasmic (**A**) and membrane-bound (**B**) PKC_*α*_ in pKS1 and *p*CO_2_ after 24 h. The lanes are the same as in Figure 6.

To further substantiate a role for PKC in the induction of proteasome expression by PIF C_2_C_12_, myoblasts were transfected with plasmids encoding constitutively active PKC-*α* (*p*CO_2_) and dominant-negative PKC-*α* (T/A)_3_ (pKS1) (Bornancin and Parker, 1996; Schonwasser *et al*, 1998), and induced to differentiate into myotubes. Myotubes transfected with *p*CO_2_ showed an increased sensitivity to PIF, as determined by the ‘chymotrypsin-like’ enzyme activity (Figure 5

) in comparison with wild-type myotubes, with a significant increase within 3 h of PIF addition (Figure 5A) persisting up to 48 h (Figure 5D). In addition, the elevation of ‘chymotrypsin-like’ enzyme activity in myotubes transfected with *p*CO_2_ greatly exceeded that in wild type at all time points. In contrast, myotubes transfected with the dominant-negative PKC_*α*_, pKS1 showed no elevation in ‘chymotrypsin-like’ enzyme activity in response to PIF at any time point (Figure 5). These results were confirmed by Western blotting of cellular supernatants for 20S proteasome *α*-subunit expression (Figure 6A

) and E2_14k_ (Figure 6B). Proteolysis-inducing factor induced an increase in both proteasome *α*-subunit expression and E2_14k_ in *p*CO_2_ but not pKS1, confirming a role for PKC in this process. The ability of PIF to activate PKC_*α*_ in *p*CO_2_, but not in pKS1, was confirmed by Western blotting (Figure 7

). The concentrations of PIF causing maximum activation of PKC_*α*_ were the same as those inducing 20S proteasome *α*-subunit expression (Figure 6A).

**Figure 8:**
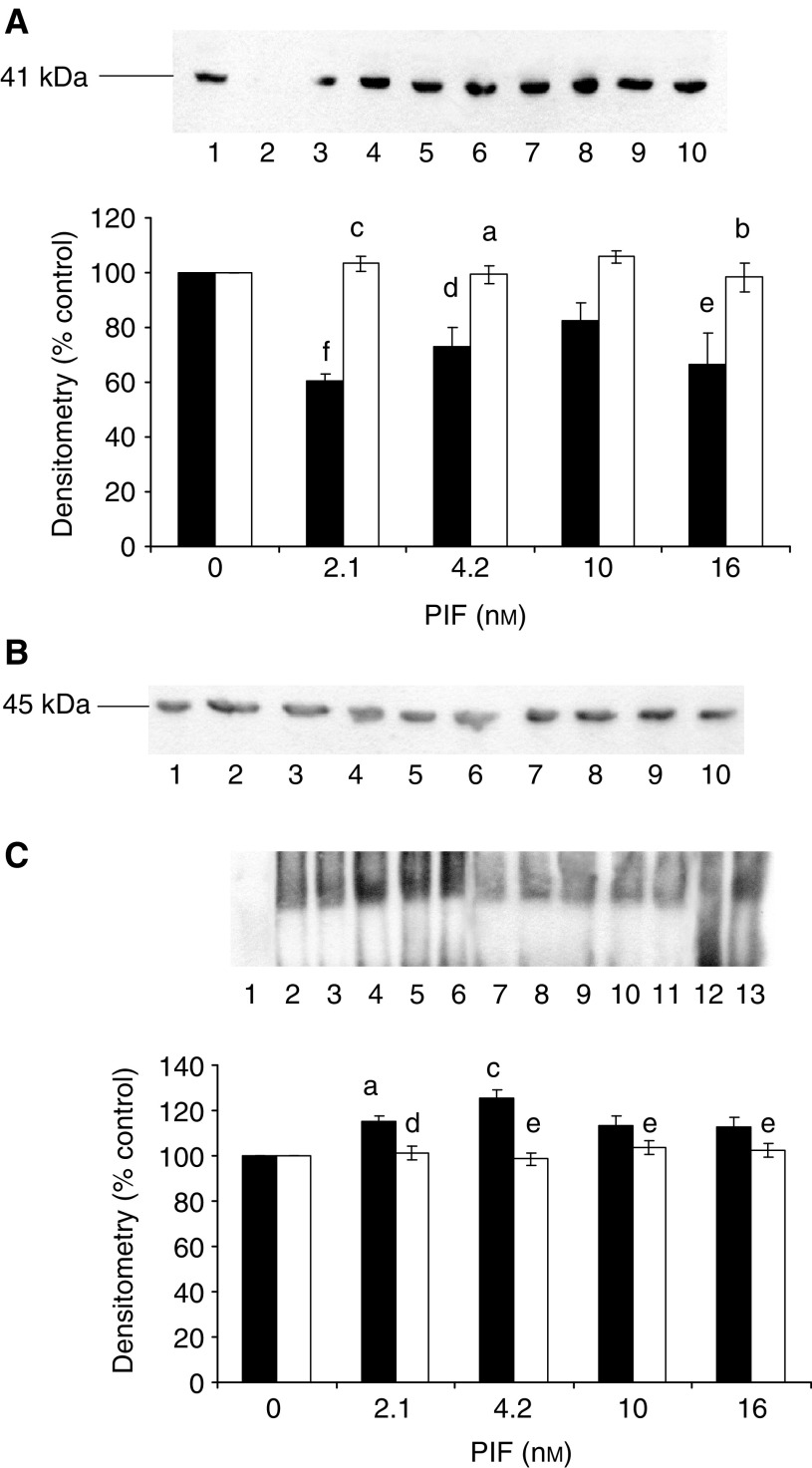
Effect of PIF and calphostin C on cytoplasmic I-*κ*B*α* (**A**) and nuclear-bound NF-*κ*B (**C**) determined 30 min after PIF addition to murine myotubes. (**A**) Myotubes were treated with 0 (lanes 1 and 6), 2.1 (lanes 2 and 7), 4.2 (lanes 3 and 8), 10 (lanes 4 and 9) or 16.8 nM PIF (lanes 5 and 10) in the absence (lanes 1–5) or presence (lanes 6–10) of calphostin C (300 nM) added 2 h prior to PIF. (**B**) Actin loading control for the blot shown in (**A**). (**C**) Nuclear levels of NF-*κ*B in myotubes in the absence (lanes 2–6) or presence (lanes 7–11) of calphostin C. Lane 1 is a negative control and lane 13 a positive control and lane 12 contains excess unlabelled NF-*κ*B. The other lanes were the same as in (**A**). The densitometric analysis is an average of three replicate EMSAs. Differences from control are indicated as ^a^*P*<0.05 and ^c^*P*<0.001, while differences in the presence of calphostin C are indicated as ^d^*P*<0.01 and ^e^*P*<0.001.

**Figure 9:**
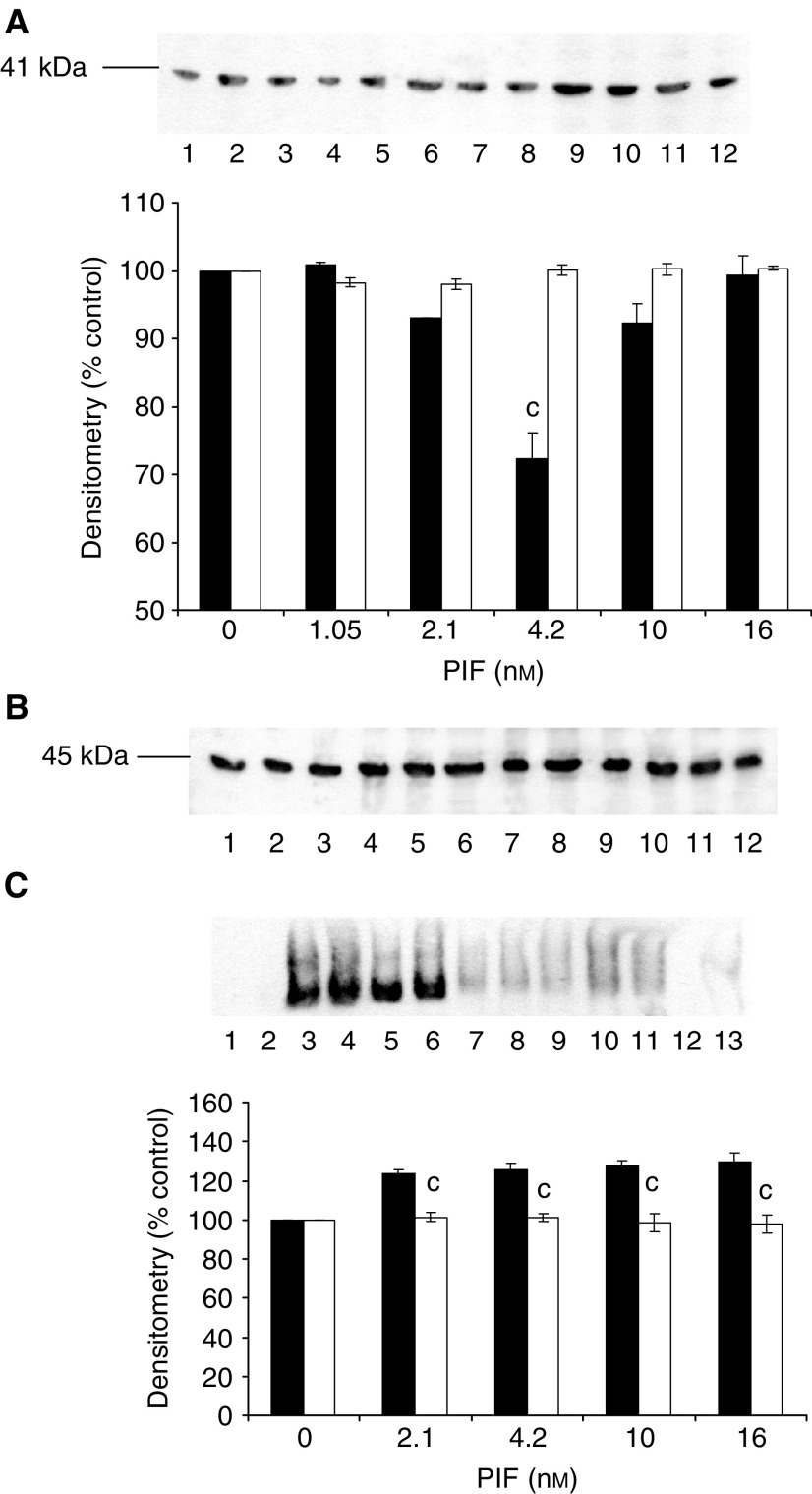
Effect of PIF on cytoplasmic I-*κ*B*α* (**A**) and nuclear-bound NF-*κ*B (**C**) determined 30 min after PIF addition to murine myotubes transfected with constitutively active (*p*CO_2_ □) and mutant (pKS1 ▪) PKC_*α*_. (**A**) Myotubes transfected with either *p*CO_2_ (lanes 1–6) or pKS1 (lanes 7–12) were treated with 0 (lanes 1 and 7), 1.05 (lanes 2 and 8), 2.1 (lanes 3 and 9), 4.2 (lanes 4 and 10), 10 (lanes 5 and 11) or 16.8 nM PIF (lanes 6 and 12). The densitometric analysis is an average of three replicate blots. Differences from 0 nM PIF are shown as ^c^*P*<0.001. (**B**) Actin loading control for the blot shown in (**A**). (**C**) EMSA of NF-*κ*B nuclear binding in murine myotubes transfected with *p*CO_2_ (lanes 2 and 6) and pK1 (lanes 7–11). Myotubes were treated with 0 (lanes 2 and 7), 2.1 (lanes 3 and 8), 4.2 (lanes 4 and 9), 10 (lanes 5 and 10) and 16.8 nM PIF (lanes 6 and 11). Lane 1 is a negative control containing the labelled probe without a nuclear extract; lane 12 contains a 100-fold excess of unlabelled NF-*κ*B probe and lane 13 is a positive control for NF-*κ*B (supplied by the manufacturers of the kit). The densitometric analysis is an average of three replicate EMSAs. Differences from control are indicated as ^b^*P*<0.01 and ^c^*P*<0.001.

We have recently shown (Whitehouse and Tisdale, 2003) that PIF-induced proteasome expression appears to require activation of NF-*κ*B. One mechanism by which PKC may function in the PIF signalling pathway is activation of IKK with subsequent phosphorylation and degradation of I-*κ*B, and translocation of NF-*κ*B from the cytosol to the nucleus (Vertegaal *et al*, 2000). Evidence for this hypothesis is provided by the following experiments. Proteolysis-inducing factor induced a decrease in cytoplasmic I-*κ*B*α* within 30 min of addition to wild-type cells (Figure 8A

), accompanied by nuclear accumulation of NF-*κ*B (Figure 8C) and this effect was completely attenuated by calphostin C (Figure 8). In addition, PIF induced a decrease in I-*κ*B*α* (Figure 9A

) and an increase in DNA binding of NF-*κ*B (Figure 9C) in myotubes transfected with constitutively active PKC_*α*_ (*p*CO_2_), but not in those containing dominant-negative PKC-*α* (pKS1). These results suggest that activation of PKC by PIF in muscle cells leads to I-*κ*B*α* degradation, nuclear accumulation of NF-*κ*B and an increased proteasome expression leading to increased intracellular protein degradation (Whitehouse and Tisdale, 2003).

## DISCUSSION

Although increased intracellular protein catabolism is a common feature of many disease states, there is little knowledge of the cellular signalling pathways involved, which may be useful in therapeutic intervention. Initial studies suggested that prostaglandin E_2_ (PGE_2_) was involved in total protein degradation in skeletal muscle, based on the demonstration in a variety of muscle types that tyrosine release was stimulated by arachidonic acid and PGE_2_ (Rodemann and Goldberg, 1982) and blocked by prostaglandin synthesis inhibitors (Strelkov *et al*, 1989). However, other studies (Hasselgren *et al*, 1990) found no evidence that total or myofibrillar protein breakdown in normal or septic muscle is regulated by PGE_2_. Studies with PIF showed that total protein breakdown was related to the release of arachidonic acid and formation of PGE_2_, but that PGE_2_ was not the eicosanoid responsible for the effect (Smith *et al*, 1999). Although the arachidonic acid was converted into a range of PGs and HETEs, only one metabolite 15-HETE alone was capable of inducing protein degradation. Further studies showed that 15-HETE induced an increase in expression of the ubiquitin–proteasome pathway, which was responsible for the initiation of protein catabolism and that this process involved the transcription factor NF-*κ*B (Whitehouse *et al*, 2003).

The present study has investigated the possibility that PKC may act as an intermediate in the PIF signalling pathway transmitting the rise in 15-HETE into activation of NF-*κ*B. The results support the suggestion that PKC plays a central role in the induction of proteasome expression by PIF and thus protein degradation. Previous studies (Smith and Tisdale, 2003) have shown that PIF induces activation of phospholipase C (PLC) as an important signalling event in inducing proteasome expression. Activation of PLC would result in the generation of diacylglycerol (DAG), which would then induce translocation of PKC from the cytosol to the membrane, resulting in the complete activation of the kinase. Indeed, PIF has been shown to induce translocation of PKC_*α*_ from the cytosol to the membrane at the same concentrations as those inducing proteasome expression. The importance of this step to the induction of proteasome expression by PIF is shown by the attenuation of this process by a range of inhibitors of PKC. In addition, myotubes transfected with a dominant-negative mutant of PKC_*α*_ also showed no induction of proteasome expression in the presence of PIF. Interestingly, myotubes transfected with constitutively active PKC_*α*_ showed an increased induction of proteasome expression compared with their wild-type counterparts, confirming the importance of this pathway in the signalling cascade. At present, it is not known which particular isoenzymes of PKC are involved in this process, or indeed whether activation of PKC occurs through production of DAG via PLC or directly through production of 15-HETE.

Attenuation of PIF-induced activation of PKC provides another control point where EPA may interfere with the signalling cascade leading to increased proteasome expression. Eicosapentaenoic acid is an effective anticachectic agent both in murine models of cachexia (Beck *et al*, 1991) and in weight-losing patients with pancreatic cancer (Barber *et al*, 1999), and effectively attenuates PIF-induced proteasome expression in murine myotubes (Whitehouse and Tisdale, 2003). Eicosapentaenoic acid inhibits both the release of arachidonic acid from membrane phospholipids and formation of 15-HETE in response to PIF (Smith *et al*, 1999), and stabilised the NF-*κ*B/I-*κ*B complex in the cytosol, preventing nuclear accumulation of NF-*κ*B (Whitehouse and Tisdale, 2003). This study shows that EPA also attenuates PIF-induced activation of PKC, which may be due to reduced generation of DAG (Sperling *et al*, 1993). In addition, DAG with an n-3 polyunsaturated fatty acid (PUFA) occupying the sn-2 position were found to be less effective in activating PKC than DAG with an n-6 PUFA, and n-3 PUFA decreased the effectiveness of activation of PKC and binding of phosphatidyl serine in the cell membrane (Terano *et al*, 1996).

Protein kinase C *α* is an upstream activator of the I-*κ*B kinase complex (IKK) (Vertegaal *et al*, 2000), which phosphorylates I-*κ*B*α* at serines-32 and -36 leading to ubiquitination and subsequent proteasome proteolysis. This suggests a mechanism by which PIF may induce degradation of I-*κ*B*α* and stimulate nuclear binding of NF-*κ*B (Whitehouse and Tisdale, 2003). Nuclear factor-*κ*B regulates the transcription of a number of genes and has been shown (Li and Reed, 2000) to be an essential mediator of TNF-*α*-induced protein catabolism in differentiated muscle cells. This study shows that degradation of I-*κ*B*α* and translocation of NF-*κ*B to the nucleus in response to PIF is attenuated by calphostin C and is not seen in myotubes expressing mutant PKC_*α*_. This suggests that PKC acts as an important mediator in activation of NF-*κ*B in response to PIF. It is not known whether NF-*κ*B acts alone or in concert with other transcriptional activators in PIF-induced proteasome expression and future studies will be aimed at identifying the role of NF-*κ*B in this process.
